# Roles of LonP1 in Oral-Maxillofacial Developmental Defects and Tumors: A Novel Insight

**DOI:** 10.3390/ijms232113370

**Published:** 2022-11-02

**Authors:** Haozhen Ma, Wanting Chen, Wenguo Fan, Hongwen He, Fang Huang

**Affiliations:** 1 Hospital of Stomatology, Sun Yat-sen University, Guangzhou 510055, China; 2 Guangdong Provincial Key Laboratory of Stomatology, Guangzhou 510080, China; 3 Guanghua School of Stomatology, Sun Yat-sen University, Guangzhou 510080, China

**Keywords:** LonP1, mitochondrial Lon protease, proteolysis, molecular chaperone, mitochondrial DNA, oral and maxillofacial oncology, CODAS

## Abstract

Recent studies have indicated a central role for LonP1 in mitochondrial function. Its physiological functions include proteolysis, acting as a molecular chaperone, binding mitochondrial DNA, and being involved in cellular respiration, cellular metabolism, and oxidative stress. Given its vital role in energy metabolism, LonP1 has been suggested to be associated with multi-system neoplasms and developmental disorders. In this study, we investigated the roles, possible mechanisms of action, and therapeutic roles of LonP1 in oral and maxillofacial tumor development. LonP1 was highly expressed in oral-maxillofacial cancers and regulated their development through a sig-naling network. LonP1 may therefore be a promising anticancer therapy target. Mutations in *LONP1* have been found to be involved in the etiology of cerebral, ocular, dental, auricular, and skeletal syndrome (CODAS). Only patients carrying specific *LONP1* mutations have certain dental abnormalities (delayed eruption and abnormal morphology). LonP1 is therefore a novel factor in the development of oral and maxillofacial tumors. Greater research should therefore be conducted on the diagnosis and therapy of LonP1-related diseases to further define LonP1-associated oral phenotypes and their underlying molecular mechanisms.

## 1. Introduction

The mitochondrial Lon protease, alternatively called LonP1, is a protease that is encoded by the nucleus but resides in the mitochondrial matrix. Over 40 years ago [1], LonP1 was studied for its powerful physiological functions, including the degradation of oxidized or damaged proteins, modulation of mitochondrial DNA (mtDNA), and maintenance of mitochondrial homeostasis. These functions enable LonP1 to mediate vital physiological processes such as cellular respiration, cellular metabolism, and oxidative stress, but also to contribute to certain diseases, including neoplasms, developmental defects, neurodegenerative diseases, and cardiac disorders. Recently, LonP1 has been found to play an essential role in the growth of and tumorigenesis in oral and maxillofacial regions. For example, patients with cerebral, ocular, dental, auricular, and skeletal syndrome (CODAS) with mutations in the *LONP1* gene exhibit unique oral and maxillofacial features, including a broad, flattened skull, collapsed midface, delayed tooth eruption, and abnormally-shaped teeth [2]. LonP1 has also been found to be markedly overexpressed in tissue samples derived from oral, head, and neck cancers, thereby contributing to their invasion, metastasis, angiogenesis, and resistance to treatment through multiple pathways [3]. Here, we review the discovery, structure, and physiological function of LonP1, with a particular focus on its impact on the oral and maxillofacial regions to provide an outlook for the future.

## 2. LonP1 Discovery, Structure and Modulation

### 2.1. LonP1 Discovery

There is a long history of research on Lon proteases. In 1946, Witkin et al. reported that mutations in *Escherichia coli (E. coli)* could alter sensitivity to radiation [4], and in 1964, Howard-Flanders et al. determined that mutations in the LON locus altered radiosensitivity [5]. In 1981, the protein encoded by the *LON* gene in *E. coli* was purified and confirmed to be an adenosine triphosphate (ATP)-dependent protease, identical to the previously described *E. coli* protease La [1]. The Lon protease was later found to be highly conserved in almost all organisms, including archaea [6], eubacteria [1], fungi (yeast *Saccharomyces cerevisiae*) [7], plants (*Arabidopsis thaliana*) [8], and animals (humans [9] and rats [10]). The Lon protease is a cytoplasmic protein in prokaryotes and a mitochondrial matrix protein in eukaryotes [11]. The evolutionary conservation of LonP1 suggests that it is necessary in organisms. For example, the knockdown of LonP1 has been found to lead to apoptosis in vitro and mouse embryonic lethality in vivo [12]. In humans, LonP1 is well distributed in tissues and particularly abundant in metabolically-active tissues, including cardiac, cerebral, hepatic, and skeletal muscle [13].

### 2.2. LonP1 Structure

**Figure 1:**
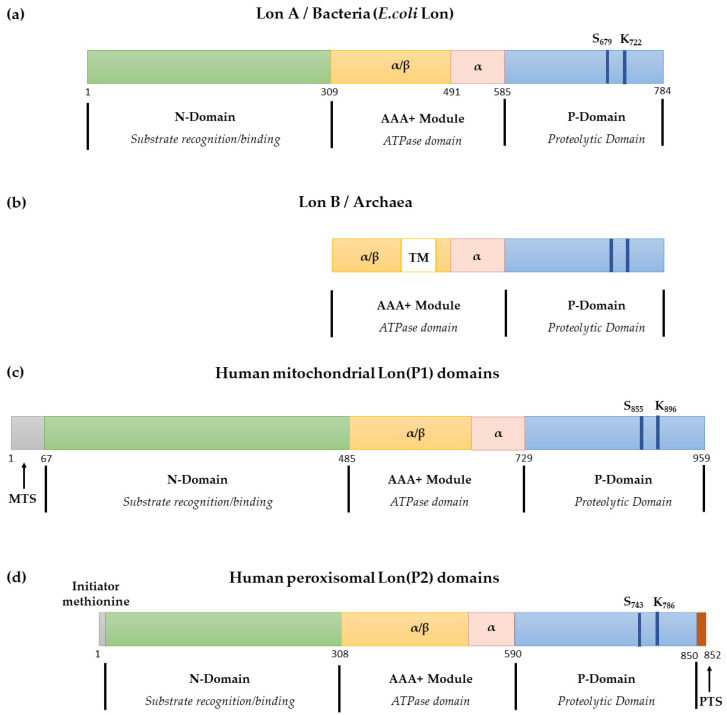
(**a**) Structure of Lon A in the primary amino acid sequence, exampled by the *E. coli* Lon. (**b**) Structure of Lon B in the primary amino acid sequence. TM, transmembrane region. (**c**) Structure of human mitochondrial Lon (P1) in the primary amino acid sequence; MTS, mitochondrial targeting sequence. (**d**) Structure of human peroxisomal Lon(P2) in the primary amino acid sequence; PTS, peroxisomal targeting sequence.

Based on their gene sequences and protein structures, Lon proteases may be divided into two types: LonA and LonB (Figure 1a,b) [14,15,16]. LonA is typified by *E. coli* Lon and includes three structural domains: a long N-terminal module that is responsible for the identification and binding of substrates, a middle ATPase domain that is engaged in ATP binding and hydrolysis, and a P domain that is capable of proteolysis at the C-terminus (Figure 1a) [16]. The ATPase domain is also known as the AAA+ module and contains α/β and α subdomains. For LonA members, the N-terminal structural domain and α subdomain in the AAA+ module are variable, while the α/β and P structural domains are highly homologous [9,17]. The N-terminal structural domain varies the most in terms of the number of amino acids and sequence. Members of the LonA family are distributed among eubacteria, fungi, plants, and animals. LonB is found in archaea [6,14]. It lacks the N-terminal structural domain but has a transmembrane structure inserted in the AAA+ structural domain (Figure 1b) [15]. LonB is highly conserved, except for its transmembrane segment. In Archaea, there is no FtsH, which is a membrane-bound ATP-dependent protease. LonB is responsible for proteolysis in archaea [14,18].

In eukaryotes, LonA family members have two isoforms: mitochondrial Lon (LonP1) and peroxisomal Lon (LonP2) [19]. Both are ATP-dependent proteases with remarkably similar structural domains, including substrate recognition, ATPase, and proteolytic domains (Figure 1d). A comparison of the amino acid sequences of LonP1 and LonP2 showed that 39.6% of the sequences were conserved [19], especially in the ATPase and protein hydrolysis structural domains. Human LonP2 is encoded by a nuclear gene derived from chromosome 16 and is directed to the peroxisomal matrix by a peroxisomal targeting sequence (PTS) after translation and assembly in the cytoplasm. The PTS is located at the C-terminus of LonP2, whereas the mitochondrial targeting sequence (MTS) of LonP1 is located at its N-terminal end (Figure 1d). In the peroxisome matrix, LonP2 scavenges oxidized or impaired proteins [20,21,22].

In this review, we focus on the structure and function of the mitochondrial Lon protease and further explore its role in oral and maxillofacial tumors and development. Below, we provide details of the structure of the *Homo sapiens* mitochondrial Lon protease (*h*LonP1), which belongs to the LonA family.

Human LonP1 is encoded by a nuclear gene on chromosome 19 and translated at the ribosome. It is then transported to the mitochondria by the MTS. After cleaving the MTS, it becomes a functional protease. *h*LonP1 is 959 aa in length (Figure 1c) and has three structural domains: an N-terminal module (aa 67–485), ATPase domain (aa 486–729), and P domain at the C-terminus (aa 730–959) [17]. The first 67 aa from the N-terminal comprise the MTS, which is responsible for targeting proteins to the mitochondria.

Before the complete 3D structure of human LonP1 was determined, its structure was mostly inferred from comparisons with the studied 3D structures of eubacterial LonP1 because the protease and ATPase structures of LonP1 are highly conserved among various species [17,23]. LonP1 is homohexameric in almost all the species that have been identified. Recent studies have shown that the LonP1 homohexamers of *E. coli* further develop into dodecamers in a head-to-head form [24]. The ratio of LonP1 dodecamers-to-hexamers is generally close to 1 but is upregulated in response to stress. LonP1 undergoes substrate selection through dodecamer formation, and dodecamers prioritize the degradation of smaller substrates.

In 2010, García-Nafría et al. determined the X-ray crystal structure of the proteolytic domain of human LonP1 and suggested its possible active form [25]. In 2016, Kereïche et al. presented the first full-length 3D structure of human LonP1 using cryo-electron microscopy [26]. During protein hydrolysis, the P structural domain of LonP1 forms a bowl-like cavity capped by an AAA+ module, while the N-terminal structural domain forms a narrow channel. It is only when adenosine diphosphate binds that the N-terminal structural domain rearranges and the entrance opens, thereby allowing substrates to enter from one side of the bowl-like cavity and proteolytic products to exit from the other side.

A recent study used cryo-electron microscopy to identify critical distinctions be-tween human and bacterial LonP1s [27]. Compared with LonP1 in bacteria, there is a second pore loop residue in *h*LonP1 that enables enhanced interactions with the translocating peptide. These enhanced interactions may strengthen the ‘grip’ of the protease on the substrate and therefore boost its potential to degrade impaired or abnormal proteins in the mitochondrial environment.

### 2.3. LonP1 Modulation

As one of the most critical proteins involved in the cellular stress response, LonP1 is biphasically regulated by stress. For instance, acute stressors such as serum starvation, heat shock, and enhanced reactive oxygen species (ROS) levels contribute to the upregulation of LonP1. Conversely, chronic or severe stressors, such as continuous oxidative stress, widespread hypoxia, and aging, can lead to the downregulation of LonP1 [18].

Chromatin immunoprecipitation (ChIP) assay confirmed the in-vivo binding of LonP1 and nuclear respiratory factor 2 (NRF-2), which is instrumental in responding to ROS and coordinating mtDNA [28]. A postulated binding site for NF-κB, which is another ROS-responsive transcription factor, is located in the LonP1 transcriptional promoter region from −2023 to −1230 [29].

LonP1 has five potential binding sites for hypoxia-inducible factor 1 (HIF-1). ChIP assays have confirmed that HIF-1 binds to LonP1 and induces its expression, which contributes to the proteolysis of cytochrome c oxidase 4-1 (COX4-1) in oxygen-deficient cells [30]. In addition, epidermal growth factor can elevate the transcriptional rate of LonP1, which stimulates carcinogenesis [31]. Protein adaptors [32], inorganic polyphosphate [33] and DNA [34] are also involved in regulating the hydrolytic activity of LonP1. LonP1 can further regulate its own activity and substrate selection by binding to itself to form a dodecamer [24].

## 3. Physiological Functions of LonP1

**Figure 2:**
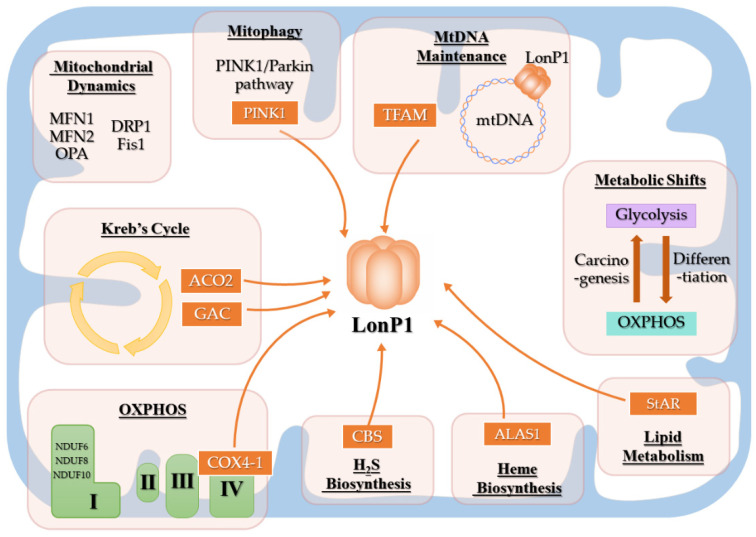
A schematic representation of the LonP1 function. The black underlined words are the functions involving LonP1, and the orange boxes with arrows pointing to LonP1 are the substrates proteolyzed by LonP1.

LonP1 functions as a protease and mtDNA regulator and modulates cellular energy metabolism, mitochondrial dynamics, mitochondrial autophagy, substance synthesis, and metabolism (Figure 2).

### 3.1. LonP1 as a Protease

LonP1 was initially characterized as a protease. Its protease behavior has been conserved throughout its evolution. The catalytic dyad necessary for proteolysis resides at residues S855-K896.

**Figure 3:**
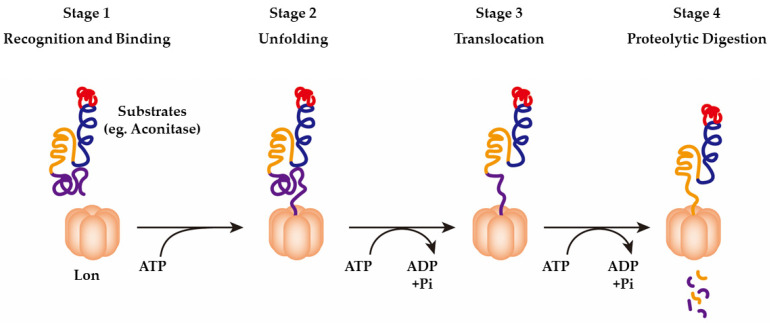
Illustration of the procedure for ATP-dependent proteolysis of substrates by LonP1, cited and modified from Venkatesh S, *Biochim Biophys Acta.* 2012 [35].

The procedure for LonP1 proteolysis is shown in Figure 3. First, the N structural domain recognizes and binds to the substrate in an ATP-independent manner (stage 1) [35]. The next step comprises the binding and hydrolysis of ATP. ATP drives an enzyme conformational transformation that facilitates the unfolding of the substrate (stage 2) and translocation of the substrate to a proteolytic zone, which is isolated from the aqueous surroundings (stage 3). Finally, the peptide bonds are cleaved, and small peptides are produced (stage 4).

LonP1 is critical for cellular function and homeostasis through the selective degradation of proteins. It predominantly degrades misfolded, redundant, damaged, and oxidized proteins that are crucial for metabolic processes, which further mediates the modulation of cellular metabolism. For example, LonP1 regulates the tricarboxylic acid cycle (TCA) through the proteolysis of two key proteins: oxidized aconitase (ACO2) [36] and misfolded glutaminase C (GAC) [37]. The impaired oxidation of ACO2 leads to its substantial accumulation, which impairs the TCA cycle and can lead to neurodegenerative diseases and a shortened lifespan. This process is reversed when LonP1 degrades oxidized ACO2. A reduction in the levels of LonP1 is closely associated with aging and mitochondrial diseases [36]. In addition, the high expression of LonP1 can cause the downregulation of ACO2 activity in tumor cells [38]. Under physiological conditions, LonP1 degrades misfolded GAC, which contributes to the proper function of the TCA cycle [39]. A previous study further found that when the toxic agent diphenylarsinic acid was added to hepatocellular carcinoma cells, *LONP1* promoted the degradation of GAC, while the accumulation of insoluble aggregates was observed in *LONP1*-knockdown cells [37]. LonP1 further contributes to the improved adaptation of cancer cells to stress in the microenvironment.

LonP1 modulates mitochondrial respiration and metabolism by selectively decomposing certain subunits of the OXPHOS complex. For example, melanoma cells with knocked-down LonP1 have reduced levels of complexes I, II, and IV, which significantly compromise cellular respiration [38,40]. In leukemic cell lines, the upregulation of LonP1 stimulates the degradation of COX4-1 and generation of COX4-2, thereby optimizing respiratory efficiency in a hypoxic state [41]. In mouse liver, heart, and lung cells in hypoxic or ischemic conditions, HIF-α has been found to induce the production of LonP1, which is responsible for COX4-1 proteolysis [30]. Sepuri et al. found that LonP1 degraded the cytochrome c oxidase subunits IVi1 and Vb under hypoxic or ischemic conditions in RAW264.7 cells [42]. In cancer cells, LonP1 regulates the electron transport chain complex of the oxidative phosphorylation pathway that includes NDUFS8, which modulates the cancer stress phenotype and promotes cancer cell survival [3,43].

LonP1 accelerates or slows the degradation of proteins associated with heme synthesis under certain conditions, thereby regulating this biochemical pathway. The normal degradation of cystathionine β-synthase (CBS) by LonP1 occurs under normoxia, but the reduced degradation of CBS under ischemic-hypoxic conditions can lead to its accumulation, which results in increased hydrogen sulfide production [44]. The primary rate-limiting enzyme in hemoglobin synthesis is 5-aminolevulinic acid synthase (ALAS-1). Excess hemoglobin can decrease ALAS-1 levels in the form of negative feedback, which is mediated by LonP1 [45].

The steroidogenic acute regulatory protein (StAR) is responsible for cholesterol production and transport. Under hormonal action, StAR is transported to the mitochondria and is involved in steroidogenesis. However, excess StAR in mitochondria can lead to mitochondrial stress. When excess levels of StAR are present in the mitochondria, NRF-2 transcription activates *LONP1*, which mediates StAR degradation to maintain mitochondrial homeostasis [28,46,47].

LonP1 is also involved in the proteolysis of PTEN-induced putative kinase 1 (PINK1), which regulates mitochondrial autophagy. In the presence of intra- and extra-cellular stress, the impairment or depletion of LonP1 can lead to the accumulation of PINK1, thereby resulting in the upregulation of mitochondrial autophagy [48,49].

### 3.2. LonP1 as a Molecular Chaperone

Several studies have suggested that LonP1 exhibits chaperone properties that are not proteolysis-dependent. It is believed that LonP1 assists in the de novo folding of proteins or in the refolding of misfolded proteins to their natural state. In *E. coli*, Lon with mutations in its proteolytic domain has been found to prevent the aggregation of unfolded proteins, which indicates that Lon may exhibit chaperone activity independent of proteolytic activity [18]. In humans, the altered expression of LonP1 does not cause changes in the protein levels of Hsp60 or mtHsp70 alone, but the knockdown of LonP1 reduces the levels of Hsp60 and mtHsp70 conjugates, as determined by immunoprecipitation assays [50,51]. Since evidence supporting the chaperone activity of LonP1 is at a modest level, the alleged chaperone activity of LonP1 may need to be researched in depth.

### 3.3. LonP1 as a Mitochondrial DNA-Binding Protein

Human LonP1 regulates mtDNA metabolism through direct or indirect binding. LonP1 can bind to both GT-rich DNA and GU-rich RNA [52,53,54]. ATP inhibits this binding, while protein substrates promote the affinity of LonP1 for nucleic acids [54]. However, it is unclear which structure of LonP1 confers its ability to bind directly to DNA or RNA [55,56]. LonP1 in *Brevibacillus thermoruber* is probably bound to DNA via the α domain of its AAA+ module [57]. However, human LonP1 mutants lacking ATPase and protease activities still retain DNA-binding activity [54]. LonP1 has also been shown to predominantly bind to regions associated with mtDNA replication and transcription. LonP1 interacts with mitochondrial nucleoids, which comprise a large class of multiprotein complexes that bind to mtDNA and promote mitochondrial genome stability, inheritance, and expression. Members of the mitochondrial nucleoids, such as mtDNA polymerase γ and Twinkle helicase, co-immunoprecipitate with LonP1 [54]. As a key to the synthesis, expression, and packaging of mtDNA, TFAM has been found to be rapidly hydrolyzed by LonP1 if not bound to DNA, as is the case with phosphorylated TFAM [58]. The amount of LonP1 negatively affects TFAM levels and mtDNA copy numbers, which ultimately modulate mitochondrial transcription [59].

### 3.4. LonP1 and Mitochondrial Function

#### 3.4.1. LonP1 and Energy Metabolism

LonP1 is involved in the regulation of vital steps in energy metabolism, such as the TCA cycle (also known as the Krebs cycle) and OXPHOS. LonP1 plays a complex and profound role in the conversion of cellular energy metabolism patterns. LonP1 directly regulates the Krebs cycle, which is a system that consists of a series of enzymatic reactions [60]. Through this cycle, the maximum amount of energy may be obtained by the complete oxidation of glucose or other substances. ACO2 and GAC, which are two key enzymes in the TCA cycle, are important substrates of LonP1 [18]. ACO2 catalyzes the first biochemical reaction in the Krebs cycle. Under normal conditions, ACO2 is enzymatically active in the TCA cycle and promotes ATP formation, whereas under oxidative stress, ACO2 is oxidatively inactivated and inhibits the TCA cycle. Oxidized ACO2 is predominantly degraded by LonP1 to reduce its adverse effects on cells [36]. GAC, which catalyzes the conversion of glutamine to glutamate, is another key enzyme in the TCA cycle. In the human hepatoma cell line HepG2, GAC assembles to form soluble tetramers under normal conditions. When stimulated by diphenylarsinic acid, GAC degrades to an unassembled state. LonP1 promotes the degradation of un-assembled GAC and prevents its aggregation to form insoluble aggregates [37]. LonP1 is crucial for the Krebs cycle, which has also been observed in *Arabidopsis thaliana*. The mitochondrial Lon protease in plants is known as Lon1. In *Arabidopsis thaliana* with the knockout of LonP1, the growth rate of shoots and roots is significantly reduced, various enzymes in the Krebs cycle are modified, enzymes related to the Krebs cycle bypass are downregulated, and the pool of organic acids in Krebs cycle intermediates is decreased [52].

LonP1 is also directly involved in the regulation of OXPHOS. A series of complexes engaged in OXPHOS is located in the inner membrane of the mitochondria and constitutes the electron transport chain. The continuous transfer of electrons in the electron transport chain generates a proton gradient, and the potential energy of this gradient serves to synthesize ATP. Cytochrome c oxidase (COX) catalyzes electron transfer and is located at the end of the electron transport chain, which is a critical regulatory point. COX has numerous subunits, of which COX4 is the key subunit governing enzyme activity [61]. COX4 has two isoforms, COX4-1 and COX4-2, and their expression is regulated by the oxygen content. In hypoxia, HIF-α is upregulated in HeLa cells, which triggers an increase in the transcription of the Lon protease that is responsible for the degradation of COX4-1. The upregulation of COX4-2 is also triggered, which increases the ratio of COX4-2 to COX4-1 and optimizes respiratory efficiency [30,62]. Goto et al. [41] revealed that hypoxia leads to the downregulation of COX4-1 and upregulation of Lon in THP-1 leukemia cells. Sepuri et al. [42] found that in hypoxic RAW 264.7 cells, two additional subunits of cytochrome c oxidase, IVi1 and Vb, were also proteolyzed by Lon. In B16F10 melanoma cells, the knockdown of LonP1 resulted in reduced tumor cell proliferation and metastasis and decreased levels of mitochondrial respiratory complexes I and III, with an overall downregulation of complex I. However, the overexpression of LonP1 resulted in enhanced tumor proliferation and metastasis and significant reductions in the levels of complexes I, II, and IV but upregulation of complex I structural proteins, such as NDUFB6, 8, 10, and 11 [38]. In oral cancer cells, Lon mediates the upregulation of mitochondrial ROS and promotion of cell proliferation by interacting with the Fe-S protein NDUFS8 in complex I [3]. The loss of LonP1 in mouse skeletal muscle leads to muscle weakness, with reduced amounts of complexes I, III, and IV in the mitochondria and a significantly-impaired respiratory capacity [63]. In Arabidopsis, the knockdown of mitochondrial Lon protease leads to the downregulation of complexes I, IV, and V [52].

LonP1 plays a complex and profound role in the rate of mitochondrial respiration and bidirectional metabolic conversion between glycolysis and OXPHOS. The knockdown of LonP1 leads to a decreased respiratory rate, impaired OXPHOS, and reduced mitochondrial function in most cases. However, the effects of LonP1 remain elusive, especially in different cellular microenvironments, such as those in inflammation, differentiation, primary carcinogenesis, and cancer metastasis.

Normally, in skeletal muscle, the knockdown of LonP1 results in a dramatic decline in the mitochondrial respiratory rate and ATP production levels [63]. In osteoarthritis, *LONP1*-knockdown chondrocytes have been found to exhibit down-regulated OXPHOS (a significant decline in the basal and maximal O_2_ consumption rate [OCR]) and further promote the progression of inflammation [64]. Analogous phenomena have been noted in cancer cells, with LonP1-knockdown glioma and prostate cancer cells displaying a lowered OCR [65,66], accompanied with the reduced proliferation and increased death of tumor cells. In addition, LonP1 knockdown reduces intracellular transport in the mitochondria of prostate cancer cells [65] and impairs the resistance to hypoxia in glioma cells [66].

LonP1 is indispensable for complicated metabolic shifts during embryonic development and cell differentiation. Murine embryos with LonP1-deficient myocardial tissue show abnormal myocardial development, heart failure, and death at birth [67]. LonP1-deficient cardiac tissue exhibits higher glycolysis and lower OXPHOS starting from E11.5, while the metabolic transition from anaerobic glycolysis to OXPHOS at E10.5 is critical for cardiac development. This indicates that the depletion of LonP1 can affect this metabolic transition [67]. However, a study by Venkatesh et al. used more developed neonatal rat ventricular myocytes to study the effects of LonP1 knockdown and found that LonP1 knockdown caused an elevated maximal OCR and enhanced fatty acid oxidation, which were possibly related to a negative feedback mechanism [68].

In cancer, LonP1-triggered energy metabolic conversion is sometimes complicated. For instance, in gastric epithelial cells, the overexpression of LonP1 has been found to boost the low multiplicity of *H. pylori* infection, thereby promoting a shift in the cellular metabolic pattern to glycolysis and further enhancing proliferation. The knockdown of LonP1 inhibited this metabolic shift [69]. In melanoma cells, both the overexpression and knockdown of LonP1 have been found to contribute to a decrease in OXPHOS and increase in glycolysis, although changes in the mitochondrial complexes varied [40]. Interestingly, LonP1 has different functions in primary and metastatic cancer cells [70]. In primary colon cancer cells, the overexpression of LonP1 results in higher maximal glycolytic activity and a higher basal and maximal OCR. In metastatic colon cancer cells, however, the overexpression of LonP1 results in a lower basal and maximal OCR [70].

LonP1 is also related to pyruvate dehydrogenase (PDH), which is a key enzyme that links glycolysis and OXPHOS. Nimmo et al. reported two siblings with mutations in the *LONP1* gene who suffered from severe hypotonia and mental retardation. Fibroblasts from both siblings had reduced PDH activity and increased intracellular lactate-to-pyruvate ratios [71].

#### 3.4.2. LonP1, Mitochondrial Dynamics, and Mitophagy

Mitochondrial dynamics refers to the continuous fission and fusion of mitochondria to achieve homeostasis and adapt to changes in the cellular microenvironment, maintain proper mitochondrial morphology, and perform mitochondrial functions [72]. Few studies have been conducted on the effects of LonP1 on mitochondrial dynamics. In osteoarthritis, chondrocytes with a knockdown of LonP1 were found to exhibit severe inflammation. Mitochondrial dynamics were comprehensively inhibited [64], with a significant downregulation of fusion proteins, such as MFN1, MFN2, and OPA, and mitofission proteins, such as DRP1 and Fis1. Mitochondria also became small, round structures or disappeared [64]. Similarly, the knockdown of LonP1 in myoblasts has been found to cause the mitochondria to become round, with the levels of Drp1 and Mfn2 being reduced [73].

Mitophagy refers to the prompt elimination of impaired and dysfunctional mitochondria to maintain proper functioning. The PINK1/Parkin pathway is important for the activation of mitophagy. In chondrocytes, the knockdown of LonP1 leads to severe mitochondrial damage, and the activation of the PINK1/Parkin pathway induces mitophagy and exacerbates osteoarthritis [64]. The knockdown of LonP1 in HeLa cells leads to an increase in unfolded proteins and accumulation of PINK1, which triggers mitophagy [49]. Considerable mitophagy has also been observed in *LONP1*-knockout mice [63]. However, Huang et al. reported different results [74]. They revealed that the hepatitis B virus X protein facilitates the translocation of LonP1 to the mitochondria while reducing cytoplasmic LonP1 levels and activating the PINK1/Parkin pathway to boost mitophagy [74]. However, a contrasting finding was reported in the case of myoblast differentiation, in which LonP1 was positively correlated with the PINK1/Parkin pathway. LonP1 may therefore promote myoblast differentiation by activating the PINK1/Parkin pathway [73].

## 4. LonP1 in the Oral and Maxillofacial Regions

### 4.1. LonP1 in Oral and Maxillofacial Tumorigenesis

#### 4.1.1. Roles of LonP1 in Oral and Maxillofacial Tumorigenesis

LonP1 appears to be upregulated in various neoplasms. LonP1 has been found to be upregulated in head and neck cancer tissues, ranking in the top 1% in silico. In the laboratory, oral cancer-derived cells and tissue samples from oral squamous cell carcinoma (OSCC) showed a significant upregulation of LonP1 compared to cells or tissues without tumors [3]. In addition to oral and head and neck cancers, LonP1 was also found to be overexpressed in cells and tissue samples from malignant lymphoma [75], cervical neoplasms [76], lung adenocarcinoma, lung cancer, malignant melanoma, breast cancer [3], bladder tumors [77], leukemia [41], glioma [13], colorectal cancer [40] and renal cell carcinoma [78].

Elevated LonP1 expression is correlated with progressive TNM staging, severe histological grading, and high cancer aggressiveness. As an independent prognostic factor for cancer, high levels of LonP1 are correlated with low survival [40,77]. In vitro, LonP1 overexpression promotes the survival, proliferation, and migration of cancer cells, thereby triggering epithelial-to-mesenchymal transition [3]. In contrast, LonP1-deficient cancer cells exhibit attenuated proliferation, increased apoptosis, decreased cellular bioenergy, and reduced drug resistance. LonP1 deficiency protects mice from chemically-induced colorectal cancer. Furthermore, triterpenoids that irreversibly suppress LonP1 activity have been found to induce apoptosis and inhibit cancer cell proliferation [79].

#### 4.1.2. Mechanisms of LonP1 in Oral and Maxillofacial Tumorigenesis

The mechanisms underlying LonP1 carcinogenesis are not yet fully understood. Several studies have shown that the mechanisms by which LonP1 promotes oral and maxillo-facial cancer are complicated, with a key step being the upregulation of ROS. ROS are closely associated with cancer [80]. They promote the induction of nuclear DNA and mtDNA lesions and contribute to cancer [81]. ROS also serve as signaling molecules that stimulate cancer cell migration and invasion and promote cancer-associated fibroblast formation in the tumor microenvironment, thereby boosting cancer growth and aggressiveness [80]. ROS even promote tumor angiogenesis. In oral cancer-derived cell lines (OEC-M1 and FADU), the upregulation of LonP1 triggers ROS production, which further promotes proliferation via Ras-ERK signaling and migration and invasion via the ERK and p38 MAPK pathways [3]. In addition, the LonP1-ROS axis stimulates inflammatory factor generation and triggers epithelial-to-mesenchymal transition, angiogenesis, and M2 macrophage polarization, thereby leading to the growth of an immunosuppressive tumor microenvironment that encourages cancer development and metastasis [82]. The LonP1-ROS axis also causes the release of mtDNA, which results in PD-L1-mediated immune escape via the interferon pathway and extracellular vesicles. Therefore, in oral cancer, mtDNA and PD-L1 in extracellular vesicles may serve as promising markers for anti-PD-L1 immunotherapy [83]. LonP1 also regulates the electron transport chain complex of the OXPHOS, NDUFS8, which modulates ROS production and promotes proliferation and invasion [36]. In addition to the aforementioned findings, other research teams have confirmed the involvement of the LonP1-ROS axis in promoting other types of cancers [65,70].

LonP1 further regulates carcinogenesis by modulating the bioenergetics of tumor cells. The knockdown of LonP1 in bladder cancer, cervical cancer, prostate adenocarcinoma, and glioma cells induces a decline in mitochondrial respiratory function with reduced ATP production [65,66,76,77], and some cells exhibit the downregulation of both OXPHOS and glycolytic pathways [76,77]. Cancer cells that are deficient in LonP1 have been found to exhibit a decreased capacity to proliferate. LonP1 has also been reported to be involved in the metabolic conversion of OXPHOS to glycolysis. This is a key feature of malignancy. Cells with oncogenic phenotypes convert their metabolic pattern from aerobic OXPHOS to anaerobic glycolysis due to the rapid glycolytic turnover, faster and greater ATP production, and availability of a large number of metabolic intermediates to accommodate rapid cell proliferation and large-scale biosynthesis [84]. During the carcinogenesis of gastric epithelial cells infected with *H. pylori*, LonP1 overexpression has been found to promote the conversion of metabolic patterns to glycolysis and the massive proliferation of cells. In contrast, LonP1 knockdown significantly attenuates glycolysis and proliferation.

LonP1 has also been found to boost vimentin and N-calmodulin levels, while E-calmodulin levels decline, implying that LONP1 may be involved in epithelial-to-mesenchymal transition [3]. The molecular chaperone activity of LonP1 is also involved in the emergence and progression of oral cancers. In oral cancer cells, LonP1 suppresses p53-dependent apoptosis under oxidative stress by stabilizing and binding to p53. LonP1 levels have been shown to correlate with cytoplasmic P53 levels in OSCC samples [51]. Recent studies have found that LonP1 affects the cancer phenotype by regulating the intracellular transport of mitochondria [65], and cell resistance to hypoxia [66].

In summary, the mechanisms by which LonP1 causes oral and maxillofacial cancers may include ROS generation, disturbed energy metabolism, the upregulation of glycolysis, resistance to hypoxia, mtDNA leakage, and mitochondrial subcellular transport.

#### 4.1.3. LonP1 as a Target for Anticancer Therapy

LonP1 plays an important role in oncogenesis, cancer progression, and cancer cell invasion. LonP1-deficient cancer cells exhibit attenuated cell proliferation, increased apoptosis, and reduced drug resistance in vitro. LonP1 deficiency has been found to protect mice from chemically-induced colorectal cancer in vivo. Therefore, the establishment of LonP1 inhibitors may be a promising antitumor chemotherapy approach.

In 2008, Bayot et al. proposed coumarinic derivatives as inhibitors of LonP1 but did not study their effects on tumors [85]. In 2010, Wang et al. found that obtusilactone A and (-)-sesamin promoted cell death in lung carcinoma through the targeted suppression of LonP1 [86]. Subsequently, 2-cyano-3,12-dioxooleana-1,9-dien-28-oic acid (CDDO) and its derivatives were found to contribute to lymphoma cell death by downregulating LonP1 [75]. In colon, liver, and breast cancer cells, CDDO induces depolarization, increases mitochondrial ROS, and alters mitochondrial morphology by targeting LonP1, thereby promoting apoptosis in cancer cells [79].

Recently, the LonP1-NCLX axis has been found to promote cisplatin resistance in oral cancer cells. An association has also been noted between NCLX and LonP1 levels in oral cancer tissue samples. Inhibitors of LonP1 and NCLX may be used as potential adjuvants to cisplatin therapy to avoid chemoresistance and enhance the curative effect in patients with oral cancer [87].

### 4.2. LonP1 in Oral and Maxillofacial Development

#### 4.2.1. Roles of LonP1 in Oral and Maxillofacial Development

In 1991, Shehib et al. [88] reported a unique case with clinical manifestations comprising delayed brain development, cataracts, flattened craniofacial bones, a fluted nasal tip, delayed tooth eruption and its abnormal morphology, unusual ear shape or compromising hearing, short stature, and delayed epiphyseal development. This condition became known as cerebral, ocular, dental, auricular, and skeletal syndrome (CODAS; MIM 600373). To date, CODAS cases remain rare, with no more than 20 reported cases worldwide. Interestingly, in 2015, two separate studies were published by two large research teams almost simultaneously. Sanger sequencing confirmed that the reported patients with CODAS had mutations in the *LONP1* gene [2,89].

The first reported case of CODAS included detailed dental information [88]. The little girl did not present with the partial eruption of her deciduous second molar teeth until she was 40 months old, whereas the eruption of other primary teeth occurred when she was between 24 and 30 months old. The occlusal surfaces of her deciduous canine and the first and second deciduous molars had an abnormal morphology, such as sunken dishes, which were prone to the retention of dental plaque. Anomalous extended cusps resulted in an open bite and multiple ulcers on the ventral and lateral sides of the tongue. Unfortunately, owing to the lack of facilities available at that time, only a written description and black-and-white photographic documentation of the dental symptoms of the patient were made (see Shebib SM, *Am J Med Genet.* 1991 [88] for details of the photographic records). For this patient, in 2015, a homozygous mutation in the *LONP1* gene (*LONP1* c.2026C > T [p.Pro676Ser]) was identified, which is located adjacent to the ATP binding pocket and may compromise LonP1’s proteolytic and ATP hydrolysis capabilities (Table 1) [2].

In addition to assessing the Sanger sequencing results from the first case, Strauss et al. [2] collected data from nine other patients with CODAS for sequencing. All of the cases presented with delayed tooth eruption and abnormal tooth morphology; however, de-tailed medical records and intraoral photographs were not available. Eight of the cases were homozygous (*LONP1* c.2161C > G [p.Arg721Gly]) and one was compound heterozygous (*LONP1* c.1892C > A/c.2171C > T [p.Ser631Tyr /p.Ala724Val]). All of the mutations were located in the AAA+ module responsible for ATP binding, which led to reduced ATP hydrolysis and impaired proteolysis (Table 1) [90]. The lymphoblastoid cell line from the patients (p.Arg721Gly/p.Arg721Gly) exhibited enlarged mitochondria with electron-dense inclusion bodies and anomalous morphology, a significant decrease in mtDNA-encoded cytochrome c oxidase subunit II, and an impaired alternate respiratory capacity, which contributed to compromised mitochondrial homeostasis and function.

**Figure 4:**
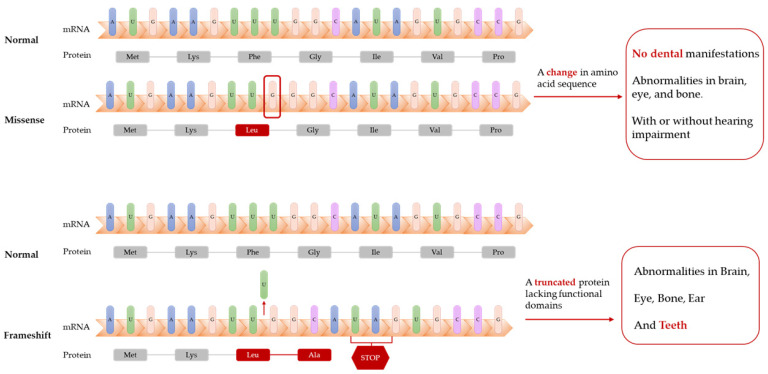
Frameshift mutations of *LONP1* gene lead to truncated proteins. Patients with these mutations have dental abnormalities (delayed tooth eruption and cusp elongation), while patients with missense mutations of *LONP1* gene might not have dental manifestations.

Dikoglu et al. [89] studied seven patients with CODAS and performed Sanger sequencing on their data. Only one patient had dental manifestations (delayed tooth eruption and cusp elongation). Unfortunately, the study only showed orthopedic radiographs and clinical photographs of the head and face of the patient but no dental photographs, which was likely due to the young age of the patient, who could not easily cooperate. The patient was heterozygous for a missense mutation and shift mutation in the *LONP1* gene, which resulted in a truncated LonP1 protein and deletion of the ATPase and protease domains (Table 1). Other patients with no dental manifestations were heterozygous or homozygous for missense mutations in *LONP1* and only exhibited mental retardation, cataracts, and epiphyseal dysplasia with or without hearing impairment (Figure 4).

The abovementioned studies suggest that biallelic mutations in *LONP1* comprise the etiology of CODAS (Table 1). Mutations occurring in the AAA+ module and shift mutations are most likely to contribute to delayed dental development and morphological abnormalities of the teeth, whereas other mutations may only cause developmental abnormalities in the brain, eyes, and bones.

#### 4.2.2. Possible Mechanisms of Action of LonP1 in Oral and Maxillofacial Development

The mechanisms by which LonP1 affects oral and maxillofacial tissue development and cell differentiation are unclear. However, insights may be gained from studies on the mechanisms by which LonP1 regulates the development of other tissues or cell differentiation. The most likely mechanism involves the LonP1-driven regulation of development through energy metabolism [67,68,73]. For example, there is a critical time point in murine embryonic heart development, E11.5, when cardiac metabolism is converted from anaerobic glycolysis to OXPHOS [67]. However, murine hearts with a deletion of LonP1 still maintain high glycolytic activity at E12.5, and myocardial development is impaired until the mice die of heart failure at birth [67]. It is believed that LonP1 is indispensable for cardiomyocytes to complete metabolic conversion, which would otherwise impair cardiac development. LonP1 is essential for myoblast myogenic differentiation. A deficiency of LonP1 inhibits myoblast mitophagy, suppresses mitofission and mitofusion, leads to mitochondrial depolarization, and thus, fails to complete differentiation [73].

## 5. Conclusions and Future Prospects

Recent studies have highlighted the central role of LonP1 in mitochondrial function. Its physiological functions include hydrolyzing oxidized and damaged proteins, acting as a molecular chaperone, binding to mtDNA, and participating in processes such as cellular respiration, cellular metabolism, and oxidative stress. Due to its important role in the mitochondria, LonP1 has been associated with multisystemic tumors and developmental disorders. Recently, new roles for LonP1 in the development of and tumorigenesis in the oral and maxillofacial regions have been identified. We present the advances made in research on the role of LonP1 in oral and maxillofacial oncogenesis and its development, underlying pathogenic mechanisms, and therapeutic applications.

The phenotypes of dental developmental malformations caused by LonP1 mutations and their molecular mechanisms of action have not yet been clarified. This is due to the fact that in previous CODAS cases, which were often first diagnosed by pediatric or orthopedic specialist physicians, medical records and imaging by oral and maxillofacial specialist physicianswere lacking. In some cases, despite detailed written records of dental developmental abnormalities, clinical photographic data were not collected, which was likely due to the young age and low cooperation of the child at the time of presentation. Such children should be followed up to obtain more imaging and photographic records from dental specialists. For this rare disease that involves multiple organs, case-based medical teams are advocated for, with pediatric, orthopedic, ophthalmologic, neurologic, and oral surgeons participating together in the diagnosis and treatment of the disease and maintaining long-term follow-up. In the future, more dentists and maxillofacial surgeons should be involved in the documentation, diagnosis, and treatment of LonP1-related diseases, and more oral scientists will be needed to further clarify the LonP1-related oral phenotype and its underlying molecular mechanisms.

## Figures and Tables

**Table 1:** Mutation details of the *LONP1* gene in CODAS patients with dental abnormalities.

Publication	Patients Amount	Homozygous/hterozygous	Mutation Type	Mutation Location	*LONP1* Variants
Strauss KA, *Am J Hum Genet.* 2015	1 patient	Homozygous	Unclear	Adjacent to the ATP binding pocket	*LONP1* c.2026C > T [p.Pro676Ser]
Strauss KA, *Am J Hum Genet.* 2015	8 patients	Homozygous	Unclear	AAA+ module	*LONP1* c.2161C > G [p.Arg721Gly]
Strauss KA, *Am J Hum Genet.* 2015	1 patient	Heterozygous	Unclear	AAA+ module	*LONP1* c.1892C > A/c.2171C > T [p.Ser631Tyr /p.Ala724Val]
Dikoglu E, *Am J Med Genet A.* 2015	1 patient	Heterozygous	Missense mutation & shift mutation	Truncated LonP1 and deletion of ATPase and P domains	c.1392G > A p.W464 *

* Represents protein truncation.

## Data Availability

Data sharing is not applicable to this article as no datasets were generated or analyzed during the current study.
